# The Role of Probiotics in Cancer Prevention

**DOI:** 10.3390/cancers13010020

**Published:** 2020-12-23

**Authors:** Katarzyna Śliżewska, Paulina Markowiak-Kopeć, Weronika Śliżewska

**Affiliations:** 1 Institute of Fermentation Technology and Microbiology, Faculty of Biotechnology and Food Sciences, Lodz University of Technology, Wólczańska 171/173, 90-924 Lodz, Poland; 2 Institute of Molecular and Industrial Biotechnology, Faculty of Biotechnology and Food Sciences, Lodz University of Technology, Stefanowskiego 4/10, 90-924 Lodz, Poland; 211706@edu.p.lodz.pl

**Keywords:** gut microbiome, probiotics, anticancer activity

## Abstract

**Simple Summary:**

Cancer is considered one of the leading causes of human mortality in the world and is the subject of much research. The risk of developing cancer depends on genetic factors, as well as the body’s immune status. The intestinal microbiome plays very important role in maintaining homeostasis in the human body. Probiotics have gained increasing medical significance due to the beneficial effect on the human body associated with the prevention and support of the treatment of many chronic diseases, including cancer in the absence of side effects. The aim of this review was to summarize the knowledge about the effect of probiotic microorganisms in the prevention of cancer. There is a lot of evidence that the use of probiotics can play an important role in cancer prevention and support anti-cancer therapies.

**Abstract:**

The gut microbiome can play important role in maintaining homeostasis in the human body. An imbalance in the gut microbiome can lead to pro-inflammatory immune responses and the initiation of disease processes, including cancer. The research results prove some strains of probiotics by modulating intestinal microbiota and immune response can be used for cancer prevention or/and as adjuvant treatment during anticancer chemotherapy. This review presents the latest advances in research into the effectiveness of probiotics in the prevention and treatment support of cancer. The described issues concern to the anticancer activity of probiotic microorganisms and their metabolites. In addition, we described the potential mechanisms of probiotic chemoprevention and the advisability of using probiotics.

## 1. Introduction

The human gastrointestinal tract is a reservoir of a complex and dynamic population of microorganisms (the gut microbiota) mainly containing bacteria (in number over 10^14^), which exerts a significant influence on the host during homeostasis and disease [1]. The presence of such a large count of intestinal bacteria means that the human body has about 10 times more prokaryotic cells than eukaryotic cells [2]. In the human intestines are found bacterial phyla: *Firmicutes*, *Bacteroides*, *Actinobacteria*, *Fusobacteria*, *Proteobacteria*, *Verrucomicrobia*, *Sinicobacteria* and *Spirochaetes* [3]. Two bacterial phyla, gram-positive *Firmicutes* (*Bacillus* spp., *Lactobacillus* spp. and *Clostridium* spp.) and gram-negative *Bacteroidetes*, predominate in human gut and represent about 90% of the bacterial population [4,5]. The gut microbiota develops and matures during the first 3 years of human life [2]. Enterotype (the type and proportion of microorganisms found in the intestines) may indirectly affect the host’s energy balance. The appropriate balance between bacterial populations ensures homeostasis of the gastrointestinal tract. However, the composition of the intestinal microbiome is susceptible to change. Thus, many factors such as improper diet, stress, gastrointestinal diseases, obesity or taking medications can lead to intestinal homeostasis disorders. As a result of imbalance of the digestive system may be proinflammatory immune responses and initiate disease processes, including cancer. Intestinal dysbiosis may be the reason for the tumorigenesis of both local gastro-intestinal cancers and tumors localized in distant sites of the body [6].

The use of probiotics has a beneficial effect on the human gut microbiome. Their main advantage is the effect on the development of the microbiota inhabiting the organism in the way ensuring proper balance between the bacteria that are necessary for a normal function of the organism and pathogens. Beneficial functions of probiotics lead to the restoration (in case of disturbance) and maintenance of intestinal homeostasis. Probiotics are live microorganisms which, when administered in adequate amounts, confer a health benefit on the host [7,8]. The sources of probiotics in the human diet are mainly silage (e.g., cabbage and cucumbers) and fermented milk products (e.g., yogurt, kefir). Probiotic microorganisms commonly used in human nutrition belong mainly to the genera: *Lactobacillus*, *Bifidobacterium*, *Lactococcus*, *Streptococcus* and *Enterococcus*. Moreover, some strains of *Bacillus* and *Saccharomyces* are used [9].

## 2. The Use of Probiotics in the Chemoprevention of Cancer

Goldin and Gorbach [10] were among the first to indicate a relationship between a diet enriched with *Lactobacillus* and a reduction in the incidence of colorectal cancer (by 37% compared to controls). The results of many in vitro studies indicate beneficial properties of probiotics in modulating the proliferation and apoptosis of cancer cells including, e.g., gastric, colonic, and myeloid leukemia cells (Table 1). Most of the in vitro studies presented were performed on human colonic cancer cells. Many researchers indicate a significant antiproliferative role and/or induction of apoptosis mus musculus colon carcinoma (HGC-27) and human colonic cancer cells (Caco-2, DLD-1, HT-29) [11,12,13,14,15] and also lowering the level of IL–8 [16] by the strain *Lactobacillus rhamnosus* GG. In addition, scientists’ reports indicate the effectiveness of probiotic microorganisms (e.g., *Bacillus*: *polyfermenticus*, *subtilis*; *Bifidobacterium*: *lactis*, *adolescentis*; *Clostridium butyricum*; *Enterococcus faecium*; *Lactobacillus*: *acidophilus*, *casei*, *fermentum*, *delbrueckii*, *helveticus*, *paracasei*, *pentosus*, *plantarum*, *salivarius*, *Lactococcus lactis*; *Pediococcus pentosaceus*, *Propionibacterium acidopropionici*, *Streptococcus thermophilus*) in reducing proliferation and/or induction of apoptosis human colonic cancer cells such as Caco-2, HT-29, SW1116, HCT116, SW480, DLD-1, LoVo (Table 1). Moreover, *Lactobacillus acidophilus* CL1285 and *Lactobacillus casei* LBC80R (in the presence of 5-FU) induced of apoptosis on human colorectal cells (LS513) [17], while *Lactobacillus acidophilus* SNUL, *Lactobacillus casei* YIT9029 and *Bifidobacterium longum* HY8001 suppressed proliferation of human colorectal (SNUC2A) and gastric carcinoma cells (SNU1) [18]. A drug that is used during chemotherapy, 5–fluorouracil (5–FU), often affects the occurrence of diarrhea. A beneficial effect of lowering cell colony formation in human colonic epithelial cells (NMC460) was found in *Bacillus polyfermenticus* [19].

Many studies on the anti-tumor effects of probiotics are carried out in animal models. The results of most of this type of research proved to be promising and indicated a potential clinical application. Research related to the effect of probiotic bacteria to animals with tumor-bearing or tumor-induced, is presented in Table 2. These results show that probiotics have anti-cancer properties. The treatment of *Bacillus polyfermenticus*, *Bifidobacterium*: *infantum, bifidum*, and *Lactobacillus: acidophilus*, *casei*, *lactis*, *plantarum*, *rhamnosus*, *salivarius* significantly inhibited the development of colon cancer in rats or mice injected with carcinogenic 1,2-dimethylhydrazine (DMH) (Table 2). In different studies, administration of probiotics such as *Pediococcus pentosaceus* or *Lactobacillus plantarum* in mice induced apoptosis and decreased the incidence of azoxymethane (AOM)-induced cancer [23,34]. The reduction the incidence of dextran sulfate sodium (DSS)-induced cancer was observed in the result of the administration in mice probiotics mixture VSL#3 [35] or *Lactobacillus plantarum* [23]. Probiotics (*L. plantarum*, *L. rhamnosus*, mixture VSL#3, *B. polyfermenticus*, *B. lactis* KCTC 5727) proved effective in the treatment of cancer induced respectively: CT26 cells injection, MNNG, TNBS, DLD-1 cells injection and in the case no injection (Table 2). Despite the promising results, these results should be interpreted with caution due to the fact that most of the tumors were induced by various chemical agents, which was quite different from the natural process of carcinogenesis.

Due to the antiproliferative role and proapoptotic effects of probiotic strains toward various carcinoma cells (in vitro studies using cell lines) and beneficial effects in animal’s model (in vivo studies), probiotics-based regimens might be used in the cancer prevention and as an adjuvant treatment during anticancer chemotherapy (Table 3).

The results of many clinical studies indicate the effectiveness of probiotics in preventing, treating and reducing the progression of several types of cancer including colorectal, liver, breast, bladder, colon, and cervical in cancer patients (Table 4). The research results indicate, among others that peri-operative administration of probiotics effectively reduces post-operative infectious complications. Infection during abdominal surgery, which is considered a factor in patients’ morbidity, can be reduced by administering probiotics to patients prior to surgery. In addition, it turns out that certain probiotic microorganisms are useful in the control of various intestinal disorders, including fever, postoperative inflammatory diseases, viral diarrhea and antibiotic or chemotherapy/radiotherapy-associated diarrhea.

## 3. Mechanism of Probiotics Action in Cancer Prevention and Therapy

**Figure 1:**
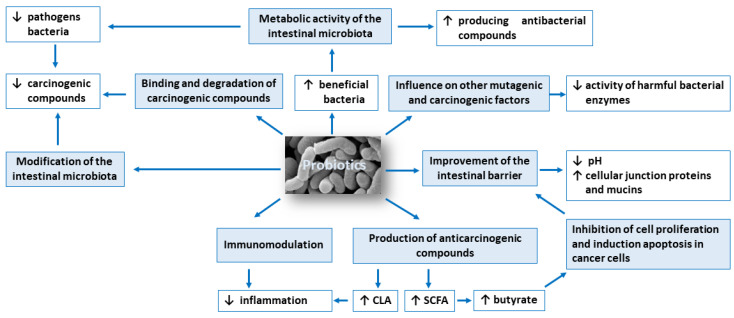
Potential mechanisms of probiotics action in the prevention of colorectal cancer development. Symbols: ↓ decrease, ↑ increase.

The anticarcinogenic activity of probiotics is based on: (1) modification of the intestinal microbiota composition, (2) metabolic activity of the intestinal microbiota, (3) production of compounds with anticarcinogenic activity, such as short-chain fatty acids and conjugated linoleic acid, (4) inhibition of cell proliferation and induction apoptosis in cancer cells, (5) influence on other mutagenic and carcinogenic factors, (6) binding and degradation of carcinogenic compounds present in the intestinal lumen, (7) immunomodulation and (8) improvement of the intestinal barrier. Figure 1 shows potential mechanisms of action of probiotics in the prevention of colorectal cancer development.

### 3.1. Modification of the Intestinal Microbiota Composition

The healthy intestinal microbiota must be properly balanced and diversified to ensure homeostasis (eubiosis). Disturbance of the intestinal microbiota balance may result in a shortage of beneficial bacteria and an excess of pathogens (dysbiosis). Moreover, dysbiosis can cause a chronic inflammation and raise the production of carcinogenic compounds which increases the risk of developing colorectal cancer [67,68].

Sobhani et al. [69] compared samples of feces from healthy people and colorectal cancer patients. Their research shows that the number of *Bacteroides* and *Prevotella* genus were significantly higher in the colorectal cancer group. In the intestinal ecosystem, several species of the *Lactobacillus* type were present in lower amounts than bacteria of the genera *Bacteroides*, *Eubacterium*, *Fusobacterium*, *Prevotella* and *Proteobacteria*. It was also found that several species of the genus *Salmonella* and *Clostridium* were present in greater numbers in patients with colorectal cancer [70]. Some strains of *Bacteroides* spp. and *Clostridium* spp. are classified as bacteria which are involved in the pathogenesis of colorectal cancer. *Bacteroides fragilis* produces enterotoxigenic toxin (fragilysin), which affects the induction of inflammatory mediators, which leads to the progression of cancer [71]. The pathogenic strain of *Escherichia coli* can synthesize several toxins, for example, cytotoxic necrotizing agent (CNF), cytolethal distending toxins (CDT), and other various virulence factors. *Streptococcus gallolyticus* and *Enterococcus faecalis* can also be connected to colorectal cancer [72,73].

The intestinal microbiota has been linked to development of the gastrointestinal cancers also by production of toxic and genotoxic bacterial metabolites that can lead to mutations by binding specific cell surface receptors and affecting intracellular signal transduction [74].

Competitive exclusion of pathogenic microorganisms by probiotics can be related to competition for nutrients and adhesion to the intestinal mucosa. There are limited nutrients available at the distal part of the colon. Probiotics compete for nutrients and grow at the expense of different intestinal microbiota [75].

Probiotic strains compete with pathogenic microorganisms for adhesion and colonization to biological membranes, forming stable, thin layers called a biofilm [76]. The microbiological biofilm develops as a result of the adhesion of microorganisms to the surface and the production of extracellular polymers that facilitate adhesion and form a structural matrix. Biofilm is characterized by structural heterogeneity, genetic diversity, complexity of interaction and the presence of extracellular substances, i.e., polysaccharides, proteins, nucleic acids, phospholipids. The secretion of these compounds is the result of adaptation to the environment. Polysaccharide polymer substances play a major role in adhesion entirely, bridging the gaps created between microorganisms in biofilm. In the result, microorganisms become focused through polymerization or association. In the first stages of biofilm formation, polysaccharides are secreted the most intensively and help first cells attach to the surface. On the other hand, proteins are first accumulated on the cell surface and then, when released, they associate on the target surface. This is usually a mixture of collagen, elastin and other proteins. They form an extracellular matrix to which microorganisms adhere [77,78].

The results of few clinical trial studies showed the beneficial effect of probiotics on the composition of gut microbiota, thus on the host by improving intestinal barrier integrity, inhibiting growth of pathogens, and reducing metabolism of pro-carcinogenic substances. Ohara et al. investigated the differences between the intestinal flora of colorectal cancer patients and healthy subjects and assessed the possibility of using probiotics to prevent colorectal carcinogenesis [60]. After ingestion of the probiotic (*Lactobacillus gasseri* OLL2716: LG21), the *Lactobacillus* detection rate increased, a decrease in the total amount of *Clostridium perfringens* was found. Moreover, fecal pH indicated acidosis, synthesis of fecal putrefaction products was inhibited, while increase in the short-chain fatty acids and isobutyric acid concentration was observed.

A deterioration of the intestinal environment was observed in the colorectal cancer patients in comparison to the healthy controls, and the intestinal environment improved when probiotics was taken, suggest the possibility of preventing colorectal carcinoma with probiotics. Kotzampassi et al. demonstrated beneficial effects of probiotics (*Lactobacillus: acidophilus*, *plantarum; Bifidobacterium lactis* and *Saccharomyces boulardii*) in patients undergoing colorectal surgery for cancer [63]. The probiotics formulation significantly decreased the risk of postoperative complications, namely mechanical ventilation, infections and anastomotic leakage.

### 3.2. Metabolic Activity of the Intestinal Microbiota

Some bacteria present in the human intestines are capable of producing carcinogenic compounds from the diet, as well as from the bile salts endogenously produced. This ability is due to the presence and activity of some enzymes, such as azoreductase, β-glucuronidase, β-glucosidase, nitrate reductase, all of which are capable of converting heterocyclic aromatic amines, polycyclic aromatic hydrocarbons, and primary bile acids into active carcinogens and synthetize aglycones, ammonia, cresols, phenols and N-nitroso compounds [79]. These metabolites have genotoxic and cytotoxic activities, which can lead to abnormal cell growth and activation of anti-apoptotic pathways in the colonocytes, thereby contributing to the development of colorectal cancer [67]. Changing the microbial metabolism by modulating the activity of these enzymes may be one of the mechanisms by which the probiotics can reduce the risk of developing colorectal cancer.

Goldin and Gorbach [80] have shown a beneficial effect of *Lactobacillus acidophilus* (strain N-2 and NCFM) on the activity of azoreductase, nitroreductase and β-glucuronidase in 21 healthy volunteers. These fecal enzymes can catalyze procarcinogens conversion to a proximal carcinogen. *Lactobacillus* strains caused a significant decline in the specific activity of the three enzymes in all subjects after ten days of feeding.

Some species of pathogenic bacteria, such as *Bacteroides*, *Clostridium*, *Escherichia coli* and *Eubacterium* exhibit higher activity of enzymes responsible for the synthesis of carcinogenic compounds. In other side, probiotic microorganisms can reduce the population of pathogenic bacteria in the intestinal microbiota and consequently reduce the intestinal production of carcinogenic compounds [81].

Metabolites of lactic acid bacteria (LAB) play an important role in controlling the intestinal microbiota. Among compounds that inhibit the development of pathogenic microorganisms, organic acids are considered to be the most important, in particular lactic and acetic acid as well as hydrogen peroxide and bacteriocins [82]. 

The antibacterial effect of organic acids can be the result of a sharp decrease in pH outside the optimum value for growth and inhibition of the biochemical activity of microorganisms by undissociated acid molecules. Lactic acid has preservative abilities, in which three factors play an important role: the effect of pH itself, the degree of acid dissociation, the specific activity of the acid molecule. Lactic acid as a weak acid in liquid environments is only partially dissociated. As a lipophilic compound in undissociated form, it can penetrate the lipid cell membrane. Inside the cell, at a higher pH of the cytoplasm, lactic acid dissociates, acidifying the cell content. By releasing potentially toxic hydroxide anions, it interferes with the proton gradient in the membranes, which is the driving force behind active transport. The acidification of the cytoplasm by lactic acid is one of the most important inhibitors of microbial growth [83]. On the other hand, acetic acid can affect cell membranes by neutralizing the electrochemical potential of the cell [84]. The presence of acetic acid can cause denaturation of intracellular proteins and a decrease in the pH value inside the cell. Acetic acid in the presence of lactic acid shows synergism in inhibiting yeast and mold growth, and may also affect the development of putrefactive bacteria, *Clostridium* and *Salmonella*. Inhibitors are also other acids, i.e., propionic, formic, benzoic, but they are produced in small quantities, and their action is based on obtaining a synergistic effect [85]. The activity of hydrogen peroxide results from strong oxidizing properties. Hydrogen peroxide can inhibit the development or kill other microorganisms that have low levels of H_2_O_2_-degrading enzymes, such as peroxidase, catalase, and superoxide dismutase, protecting against oxidation of disulfide bridges in cellular proteins. In vitro studies confirm the inhibition of various bacteria such as *Staphylococcus aureus*, *Salmonella* Typhimurium, *Escherichia coli*, *Clostridium perfringens*, *Clostridium butyricum* and *Pseudomonas* spp. by hydrogen peroxide [86,87]. On the other hand, it should be emphasized that hydrogen peroxide can also have an effect on all strict anaerobes, including beneficial gut bacteria.

Furthermore, probiotics produce bacteriocins, which are deadly or bacteriostatic on sensitive microorganisms, affecting the cell membranes of bacteria having receptors capable of attaching them. Bacteriocins can cause: a portion of the bacterial cytoplasmic membrane that leads to the dissipation of the transmembrane potential and induces the leakage of K + ions, ATP and amino acids from the cytoplasm of attacked cells; cell lysis and interfering or inhibiting the synthesis of DNA, RNA and proteins (act as DNAase or RNAase) [88,89]. The bacteriocins produced by probiotics e.g., are bifidocin B produced by *Bifdobacterium bifidum*, nisin from *Lactococcus lactis* and lactacin B from *Lactobacills acidophilus*. Bifidocin B, which is produced by *Bifidobacterium bifidum* NCFB 1454, exerts a strong inhibitory activity against several pathogenic bacteria, including *Salmonella* Typhimurium SL1344 and *Escherichia coli* C1845 [90,91]. On the other hand, Drissi et al. explored the role of bacteriocins may have in the GIT. In a genome mining research 641 genomes (307 whole genomes and 334 draft genomes) from microorganisms in the human gut were received. The genomes represented 199 bacterial genera, including *Lactobacillus*, *Streptococcus*, *Clostridium*, and *Bacillus*. Of the 317 bacteriocins, 175 were from *Firmicutes* (which includes LAB), 79 from *Proteobacteria*, 34 from *Bacteroidetes*, and 25 from *Actinobacteria*. The authors suggested that bacteriocins in the GIT may have low levels of antimicrobial activity and may thus not have such a drastic effect on microbial populations. However, it was also suggested if bacteriocins play a lesser role in population dynamics, they may have a greater role to play in quorum sensing, or possibly in host immune modulation [92].

### 3.3. Production of Compounds with Anticarcinogenic Activity, Such as Short-Chain Fatty Acids and Conjugated Linoleic Acid

Short-chain fatty acids (SCFA) are organic acids consisting of 1–6 carbon atoms in the aliphatic chain, including acetic, propionic, butyric, valeric and caproic acid. SCFAs are the main and final products of the metabolism of light-living bacteria large intestine [93]. The daily production of SCFAs in the large intestine of a healthy person should range between 300 and 400 mM, and the total concentration of these acids in the intestinal lumen should be 60–150 mM [94,95]. The molar ratio of acetate, propionate and butyrate produced in the colon is 60:25:15, respectively, but these proportions may be subject to modulation and change [96]. Although their daily production varies around 300 mM, only 10 mmol per day are excreted. This is because SCFAs are absorbed and targeted in the colon at a concentration of 6 to 12 μmol/cm-2/h [97]. SCFAs are a source of energy for colonocytes and promote acidosis and apoptosis of cancer cells, thus promoting an acidic environment that inhibits the formation of high levels of secondary bile acids. Butyric acid helps regulate the balance between proliferation, division, and apoptosis of colonocytes. About 70–90% butyrate is metabolized by colonocytes. The occurrence of that acid in the feces of healthy people is in larger amounts compared to colorectal cancer patients. In addition, it is assumed that a reduction in the 1 µg/L butyrate concentration in stools increases the risk of colon cancer by 84.2%. However, when the concentration of acetic acid is reduced by 1 µg/L, the probability of developing adenoma increases by 71.3% [70].

SCFAs are naturally produced by the bacteria that compose the intestinal microbiota. However, the amount produced may not be sufficient for inhibiting the development of colorectal cancer. Thus, consumption of probiotics may contribute to the increase of the daily production of SCFAs. The presence of SCFAs in the lumen of the large intestine can inhibit the growth of pathogens, e.g., *Salmonella* Typhimurium in the presence of propionate and butyrate, inhibits the expression of invasive genes encoding Salmonella SPI-1 pathogen islands and prevents attack on cells in tissue culture in vitro [98].

**Figure 2:**
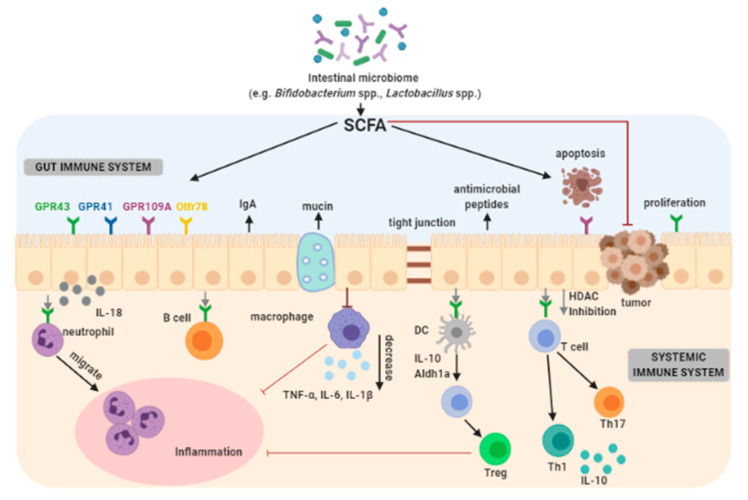
The role of short-chain fatty acids (SCFAs) in regulation of gut and systemic immunity [99,100]. SCFAs are produced by intestinal microbiome in the fermentation of nondigestible carbohydrates (NDCs), undigested food fiber, or resistant starch (RS). SCFAs regulate intestinal barrier function by inducing intestinal epithelial cell secretion of interleukin-18 (IL-18), mucin (MUC2), antimicrobial peptides, and upregulating the expression of tight junction. Moreover, SCFAs regulate the T cell function through G-protein-coupled receptors (GPR41, GPR43, GPR109A), Olfr78 receptor signaling, and through inhibition of histone deacetylase (HDAC) which affects inhibition of nuclear factors (nuclear factor-κB; NF-κB). SCFAs play an important role in inducing neutrophils migration to inflammatory site and enhancing their phagocytosis. Moreover, SCFAs inhibit intestinal macrophage production of proinflammatory cytokines (e.g., IL-6, IL-8, IL-1β and TNFα), and possibly induce intestinal IgA production of B cells. SCFAs affect the differentiation of regulatory T (Treg) cells and the production of interleukin-10 (IL-10). The direct impact of SCFA as well as SCFA regulation of dendritic cells (DCs) are mediated in the differentiation of T cells. SCFAs regulate the generation of Treg, Th1, and Th17 in different cytokine environment. SCFAs promote apoptosis and suppress proliferation of tumor cells resulting in inhibiting the carcinogenesis. Abbreviations: Aldh1A2—aldehyde dehydrogenase 1A2. Symbols:→ activation; →inhibition.

Butyric acid can contribute in the prevention of colorectal cancer because it is capable of improving the intestinal barrier through the increase in mucus production, and in the proliferation of healthy cells (Figure 2).

Butyrate has the ability to decrease the production of inflammatory cytokines by: inhibiting the activation of nuclear transcription factor kappa B, regulating the activity of proteins involved in apoptosis (Bcl-2, Bak, and caspases 3 and 7), increasing the immunogenicity of tumor cells, suppressing cyclooxygenase (COX)-2 activity, increasing the activity of the antioxidant enzyme glutathione S-transferase (GST) and inhibiting the deacetylation of histones. These effects can result in silencing or regulation of genes involved in the control of cell cycle proliferation, differentiation, and apoptosis [101].

SCFAs have immunomodulatory functions that affect the inflammatory response in selected cases by interacting with receptors that are coupled to G protein in the intestine. G protein is a heterotrimeric guanidine nucleotide binding protein. Neurotransmitters or chemokines transmit cell signals with the participation of G protein [102].

Conjugated linoleic acids (CLAs) are isomers of linoleic acid (LA) in which the carbon pairs involved in the formation of double bonds are adjacent to each other. The most commonly found CLA isomers in nature are the cis-9, trans-11 and the trans-10, cis-12. Both isomers are possessing effect against the spread of colon cancer cells [103].

CLAs have influence on the expression of genes involved in the apoptosis process (Bcl-2, caspase 3 and 9) and the cellular response to cell growth factors. In addition, CLAs are able to suppress the production of eicosanoids in colonocytes in two ways. The first consists of the replacement of arachidonic acid in the cell membranes by CLAs, and the second is the result of the interference of CLAs in the activity of the cyclooxygenase and lipoxygenase enzymes, which are responsible for the synthesis of eicosanoids [104].

According to Ewaschuk et al. some species of probiotic bacteria, such as *Lactobacillus* (*acidophilus*, *casei*, *delbrueckii*, *plantarum*), *Bifidobacterium* (*breve*, *infantis*, *longym*), *Propionibacterium freudenreichii* and *Streptococcus salivaris* subsp. *thermophilus* are capable of producing CLAs from linoleic acid [105]. This fatty acid is produced in the distal ileum by bacteria and can be absorbed by or interact with the colonocytes in the intestinal lumen, thus exerting its beneficial effects. 

### 3.4. Inhibition of Cell Proliferation and Induction Apoptosis in Cancer Cells

**Figure 3:**
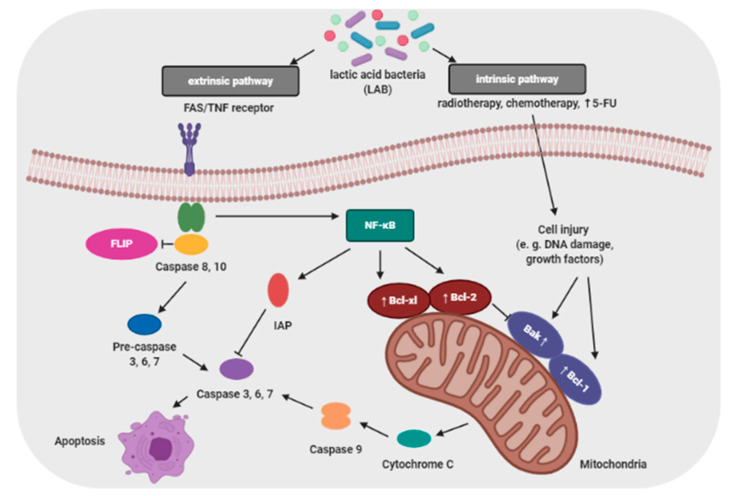
Potential mechanisms of action of lactic acid bacteria in pathways of apoptosis [50,107]. The extrinsic pathway is initiated by FAS ligand-induced activation of death receptors on the cell membrane (e.g., TNF—tumor necrosis factor). The intrinsic pathway is induced radiotherapy and chemotherapy. The mitochondrial localization and activation of Bax and Bak are required in the intrinsic pathway. However, it can be prevented by pharmacologic inhibitors or anti-apoptotic Bcl-2 family proteins. Lactic acid bacteria can enhance the apoptosis induction through 5-fluorouracile (5-FU) that can induce the activation of autophagic cell death promoted by the induction of Beclin-1 and GRP78 or Bcl-2 and Bak. Moreover, LAB may act to prevent cancer via downregulating NF-κB-dependent gene products which regulate cell survival (Bcl-2, Bcl-xl) and proliferation (Cox-2, cyclin D1). Symbols: →activation; →inhibition.

Proliferation and apoptosis of cancer cells determine the rate of cancer development. During the cancer development process, these cells proliferate more than undergo apoptosis. Probiotics that are able to modulate the cellular proliferation and apoptosis are of great interest because cancer cells would be eliminated less aggressively, and apoptosis brings no damage to the neighbor cells and does not cause inflammation [106]. The apoptosis signaling pathways can be activated by probiotic bacteria (e.g., lactic acid bacteria; LAB) through a mitochondria-dependent (intrinsic) and a death receptor–dependent, mitochondria-independent (extrinsic) pathway (Figure 3) [50].

Baldwin et al. evaluated the antiproliferative activity of *Lactobacillus acidophilus* and *Lactobacillus casei* against LS513 gastric cell lines through cellular apoptosis [17]. Hwang et al. demonstrated that probiotic induced apoptosis in gastric cancer cells (KATO3) by inhibiting NF-κB and mTOR-mediated signaling [108]. Cousin et al. demonstrated that probiotic bacteria induce chromatin condensation, apoptotic bodies, DNA fragmentation, caspase activation, inactivation of mitochondrial trans-membrane potential and cell cycle arrest [27]. Probiotic strains such as *Lactobacillus reuteri* have been reported to influence hematological cancers, which enhanced TNF-induced apoptosis in human chronic myeloid leukemia derived cells [31]. 

The increased incidence of apoptosis of cancer cells induced by probiotics has been attributed to SCFAs, particularly butyrate, which are able to induce epigenetic changes, paralyze the cell cycle, and stimulate the expression of proapoptotic genes. Relationship exist between the amount of SCFAs in the feces and cell proliferation in the colonic crypts [109].

Probiotic *Lactobacillus* spp. induced selective genotoxic, cytotoxic, pro-apoptotic effects on leukemia and colon cancer cell lines, and as anti-inflammatory effects on macrophage cells at the molecular level. 

In vitro experimental evidence suggests that probiotics used for their anti-cancer activity operate via a process of genotoxicity and cytotoxicity against tumor cells. Liu et al. explored the effects of *Lactobacillus casei* 01 on 4-nitroquinoline Noxide (4-NQO) induced genotoxicity and colon cancer cell line (HT29) [110]. Nami et al. reported that the metabolites from *Lactobacillus acidophilus* 36 YL exhibited the most potent cytotoxic effect against human cervical cancer cell lines (HeLa) and colorectal cancer cell lines (HT-29) [111].

Since in vitro studies using cell lines indicated that probiotics had proapoptotic effects on carcinoma cells probiotics-based regimens might be used as an adjuvant treatment during anticancer chemotherapy [17,31,110,112].

### 3.5. Influence on Other Mutagenic and Carcinogenic Factors

Probiotics may have influence on the other mutagenic and carcinogenic factors, thus contribute to the prevention of cancer. They are able to change the activity of some enzymes involved in the cellular detoxification process, preventing the activity of free radicals and carcinogenic substances.

Glutathione S-transferase (GST) is an antioxidant enzyme with detoxifying activity, which inactivates the carcinogens compounds such as reactive oxygen species (ROS) or xenobionts. GST role is the protection of DNA against oxidative damage, which may lead to mutations, and in consequence, favor carcinogenesis. GST gene polymorphisms may affect the functioning of the encoded enzymes, exerting an effect on the level of DNA damage, and therefore may have an indirect influence on the risk of the development of cancer [113]. Probiotics are able to increase the activity of this enzyme through the action of butyrate, which could change the status of histone acetylation, thus increasing the expression of GST [114].

The bacterial enzyme, β-glucuronidase, with broad substrate specificity can hydrolyze a number of glucuronides, causing the release of carcinogens into the colon, including PAH (e.g., benzo[a]pyrene), an important risk determinant for colorectal cancer [49]. β-glucosidase, breaks down plant glycosides cycasin in the colon, generating aglycones, which are carcinogens [115]. Nitroreductase reduces the N-nitro compounds (e.g., nitrobenzenes) to amines, which are usually mutagenic and carcinogenic, nitrate reductase participates in the generation of highly toxic and carcinogenic nitrite. Nitroreductase activity is significantly higher in colon cancer patients than healthy [116]. Commensal bacteria *Bacteroides fragilis*, *Eschericha coli* and *Clostridium* have exhibited high activities of these pro-carcinogenic enzymes (β-glucosidase, β-glucuronidase, nitroreductase). Studies have also demonstrated *Bacteroides fragilis Clostridium* and *Escherichia coli* to be over-presented in colon cancer patients [117]. 

Probiotics modulate the bacterial enzyme activity in the colon by a combination of mechanisms including: (1) competitive exclusion of pathogenic microflora, (2) ability of synthesizing antibacterial substances that inhibit the growth of other microorganism and (3) ability to generate acids that may lower the pH of the colon [118].

Studies have demonstrated that probiotics are able to reduce the activity of above mentioned procarcinogenic enzymes. *Lactobacillus rhamnosus* GG and *Lactobacillus acidophilus* treatment led to a significant reduction in β-glucuronidase activity, while the *Lactobacillus casei* or *Lactobacillus plantarum* decreased the nitroreductase activity in rats [119]. In different studies *Lactobacillus acidophilus* KFRI342 inhibited β-glucuronidase and β-glucosidase activity [43]. *Bifidobacterium longum* was also found to lower these two enzyme activities in healthy rats [120]. Clinical trials showed similar inhibitory effects of probiotic on these carcinogenic enzymes. For instance, studies showed demonstrated that *Bifidobacterium* species reduced the β-glucuronidase activity in human intestine [121], *Lactobacillus rhamnosus* GG β-glucuronidase and nitroreductase [122]. Furthermore, *Bifidobacterium adolescentis* SPM1207 and SPM0212 reduced intestinal β-glucuronidase and β-glucosidase, as well as tryptophanase and urease, producers of putrefactive products linked to higher incidence of colorectal cancer, such as indoles and ammonia [32,120].

### 3.6. Binding and Degradation of Carcinogenic Compounds Present in the Intestinal Lumen

Carcinogenic compounds may bind to the cell wall of some probiotic bacteria. This to be associated with the occurrence of cationic exchange between the carcinogenic compounds and the peptidoglycan present in the cell walls of some probiotic microorganisms. Thus, carcinogenic compounds would be eliminated together with the bacteria through the feces [123].

Studies have shown, that *Bifidobacterium longum*, *Lactobacillus acidophilus* and *Streptococcus salivarius* strains could bind and cause the release in feces of heterocyclic amines and mutagens such as: 2-amino-3,4-dimethylimidazo [4,5-f] quinoline (MeIQ), 2-amino-3-methyl-3H-imidazo [4,5-f] quinoline (MHIQ), and 5-phenyl-2-amino-l-methylimidazo [4,5-f] pyridine (PhMIP), 3-amino-1-methyl 5 h pyrido[4,3-b] indole acetate (TrpP2) [124,125].

Rowland and Grasso studied in vitro the effect of intestinal microorganisms (of the genera *Lactobacillus*, *Bifidobacterium* and *Streptococcus*) to dimethyl-nitrosamine and showed that bacteria of the genus *Lactobacillus* most actively degraded these substances [126]. The amine was transformed into its precursor, dimethylamine, as well as to other volatile metabolites. However, Morotomi and Mutai showed a high ability of *Lactobacillus casei* to detoxify mutagenic heterocyclic amines [127]. They studied effects of live and heat-inactivated bacteria, and only live showed such ability. This may indicate that live bacteria produce metabolites or catalyze reactions that lead to amine detoxification.

The literature also shows that probiotics may have the ability to detoxify mycotoxins that may have carcinogenic properties [128,129]. El-Nezami et al. demonstrated that 5-week supplementation of probiotics reduced the urinary excretion of aflatoxin B(1)-N(7)-guanine, a marker for hepatocyte carcinogenesis, and synbiotic consumption for 12-week significantly reduced colorectal cancer risk [49,128].

### 3.7. Immunomodulation

Microorganisms of the intestinal microbiota are the main factors stimulating the immune system, which is a condition for the development of lymphoid structures of this system. The immunomodulatory activity of the intestinal microbiota, including probiotic bacteria, is based on three seemingly opposite phenomena: (1) inducing and maintaining a state of immune tolerance to environmental antigens (food and inhalation), (2) induction and control of immune responses against bacterial and viral pathogens and (3) inhibiting autoimmune and allergic reactions.

Probiotic immune stimulation is also manifested in increased production of immunoglobins, increased activity of macrophages, lymphocytes and stimulation of γ-interferon production. The components of the cell wall of lactic acid bacteria stimulate the activity of macrophages, which due to the increased amount of free oxygen radicals and lysosomal enzymes are able to quickly destroy microbes. Probiotic bacteria also have the ability to stimulate cytokines by immunocompetent gastrointestinal cells [130]. Some probiotics can also affect the immune response through the activation of phagocytes and contribute to the maintenance of the state of vigilance, which can eliminate cancer cells in their early stages of development [131].

The adaptive immune response depends on T and B lymphocytes, which are specific for particular antigens whereas innate immune system responds to common structures called pathogen-associated molecular patterns (PAMPs) shared by the vast majority of pathogens. The primary response to pathogens is triggered by pattern recognition receptors (PPRs), which bind PAMPs. The best-studied PPRs are Toll-like receptors (TLRs). TLRs are transmembrane proteins expressed on various immune and nonimmune cells, such as B cells, natural killer cells, macrophages, dendritic cells (DC), epithelial cells, fibroblasts and endothelial cells. Probiotics can suppress intestinal inflammation via the downregulation of TLR expression, secretion of metabolites that may inhibit TNF-α from entering blood mononuclear cells, and inhibition of NF-κB signaling in enterocytes [132].

In the case of colorectal cancer, the proinflammatory cytokines IL-1β, IL-6, IL-8, IL-12, IL-17, and tumor necrosis factor-α (TNF-α) can be associated with the development of cancer [79]. Probiotics are increase the production of anti-inflammatory cytokines and decrease the production of proinflammatory cytokines, and the development of the colon cancer cells can be delayed. In addition, probiotics may decrease the expression of COX-2, an enzyme that catalyzes the production of prostaglandins from arachidonic acid, which has been linked to an increased risk of developing colorectal cancer because it stimulates cell proliferation and the proinflammatory process [67,133].

Probiotic microorganisms also regulate the activity of natural killer (NK) cells. The use of the probiotic *Lactobacillus casei* subsp. *casei* in combination with dextran enhances the efficiency of NK cell activity. This property may be linked to intestinal epithelial cell production of IL-15, an important cytokine for NK cells [134]. It was found that *Lactobacillus casei* Shirota enhanced NK cell activity which was correlated to an IL-12 production, cytokine implicated in NK cells activity [135].

### 3.8. Improvement of the Intestinal Barrier

The microorganisms of the intestinal microbiota may change the intestinal barrier and permeability. Some of probiotics are able to reduce intestinal permeability because they can modify components of the intestinal barrier, such as intracolonic pH, the production of mucins and the cellular junction proteins [136].

Lower intracolonic pH values (more acidic) inhibit the proliferation of pathogenic and putrefactive bacteria, as well as the activity of bacterial enzymes responsible for the production of carcinogenic compounds [137]. In several in vitro studies has been confirmed that probiotic bacteria inhibit the growth of gram-negative pathogenic microorganisms. This growth-inhibiting activity has generally been attributed to the fact that probiotic strains lower the pH and/or produce lactic acid. For example, strains of *L. acidophilus*, *L. casei* subsp. *rhamnosus*, and *Lactobacillus bulgaricus* inhibited the growth of clinical isolates of *Helicobacter pylori* [138,139] and *L. casei* subsp. *rhamnosus* strain Lcr35 reduced the growth of enteropathogenic and enterotoxigenic *Eschericia coli*, and *Klebsiella pneumoniae* [140]. It was shown that inhibition occurred when the pH of the incubation medium was acid and that no growth inhibition occurred when the pH of the incubation medium was neutral.

Probiotics can regulate intracellular pH, which allows them to survive and maintain metabolic activity at a relatively low pH environment. This is possible due to the rapid secretion of lactic acid from the cells, which causes the cytoplasmic pH in the cells to become more alkaline than the living environment. In addition, biological membranes are relatively impermeable to protons and lactic acid molecules. This creates a pH gradient (ΔpH) between the cytoplasm and their living environment.

The mucin protective layer, which is produced by goblet cells are gel-forming glycoproteins act as lubricants and as a protective barrier between the body and the external environment. At least nine human mucin (MUC) genes have been identified, and MUC1, MUC2, MUC3, MUC4 and MUC5AC are expressed in the human colon. MUC2 is the major gel-forming mucin of the small and large intestines and is the main structural component of the mucus gel. The carcinogenic process decreases the production of mucins and makes their composition less glycosylated. Some probiotics are able to increase the production of mucins by goblet cells through the upregulation of the MUC genes, mainly MUC2. For in vivo studies, Wistar rats were orally administered the probiotic VSL#3 (*Lactobacillus: plantarum*, *acidophilus*, *casei*, *delbrueckii* subsp. *bulgaricus*, *Bifidobactrium*: *infantis*, *breve*, *longum* and *Streptococcus salivarius* subsp. *thermofilus*) on a daily basis for seven days. Probiotic significantly stimulated colonic mucin MUC (by 60%) secretion and MUC2 gene expression, (up to 5 times) however, MUC1 and MUC3 gene expression were only slightly elevated [141].

The inflammatory and carcinogenic processes increase intestinal permeability, mainly because they change the structure and expression of the cellular junction proteins, which makes colonocytes adhere to each other. These proteins are found mostly in the apical region between the colonocytes and are formed by a complex of transmembrane proteins that bind to the colonocyte cytoskeleton through the junction transmembrane proteins, forming the tight junctions [76]. Probiotics can reduce intestinal permeability because they can change the distribution of cell junction proteins and improve the distribution of these proteins throughout the colonic epithelium, making it more continuous. n the small intestine of healthy subjects, administration of *Lactobacillus plantarum* WCFS1 induced changes in the epithelial tight junctions, resulting in increased staining of the scaffold protein zonula occludens-1 and the transmembrane protein occluding. *Lactobacillus plantarum* induced translocation of zonula occludens-1 to the tight-junction region was also seen in an in vitro model of the human epithelium, and this significantly protected against chemically induced disruption of the tight junction and the associated increase in epithelial permeability. The mechanism was shown to be dependent on Toll-like receptor 2 signaling and highlights the homeostatic role of innate signaling pathways in maintaining human intestinal epithelial barrier functions [142].

## 4. Conclusions

Probiotics have gained increasing medical significance due to the beneficial effect on the human body associated with the prevention and support of the treatment of many diseases in the absence of side effects. Due to the potential mechanisms of action presented in this paper, probiotic microorganisms can have a beneficial effect both locally and on the body as a whole. The probiotic properties of microorganisms are a strain trait. There is a lot of evidence that the use of probiotics can play an important role in cancer prevention and support anti-cancer therapies. As a result of laboratory research, many promising results were obtained, which indicate the antitumor effect of probiotics. However, the presented research results confirm the effectiveness of probiotics only for potential prevention of cancer or as adjuvant treatment during anticancer chemotherapy. Clinical trials are still not enough to unambiguously confirm the potential of probiotic microorganisms in this regard. Therefore, it is very important and desirable to continue research on the anti-cancerogenic properties of specific probiotic strains and their mechanisms of action (especially during treatment). In addition, a randomized, double-blind, placebo-controlled clinical trial should be conducted to obtain approval from the medical community and validate the potential of probiotics as an alternative cancer therapy.

## Figures and Tables

**Table 1:** Effects of probiotic microorganisms on cancer cells in in vitro studies.

Reference	Probiotic Strain	Cell Line	Effect
[20]	*Lactobacillus pentosus* B281 *Lactobacillus plantarum* B282	Caco-2 and HT-29	↓ Cell proliferation, Cell cycle arrest (G1)
[21]	*Lactobacillus casei* ATCC 393	HT29 and CT26	Induction of apoptosis
[22]	*Lactococcus lactis* NK34	HT-29, LoVo, AGS	>80% ↓ Cell proliferation
[23]	*Bacillus polyfermenticus* KU3	LoVo, HT-29, AGS	>90% ↓ Cell proliferation
[24]	*Clostridium butyricum* ATCC *Bacillus subtilis* ATCC 9398	HCT116, SW1116, Caco-2	↓ Cell proliferation
[15]	*Lactobacillus plantarum* A7 *Lactobacillus rhamnosus* GG	Caco-2, HT-29	↓ Cell proliferation
[25]	*Lactobacillus kefiri* P-IF	MDR	Induction of apoptosis
[26]	*Pediococcus pentosaceus* FP3 *Lactobacillus salivarius* FP25/FP35 *Enterococcus faecium* FP51	Caco-2	↓ Cell proliferation, Activation of apoptosis
[27]	*Propionibacterium freudenreichii* ITG P9	HGT-1	Induction of apoptosis
[13]	*Lactobacillus paracasei* IMPC2.1 *Lactobacillus rhamnosus* GG	DLD-1, HGC-27	↓ Cell proliferation, Induction of apoptosis
[12]	*Lactobacillus rhamnosus* GG *Bifidobacterium lactis* Bb12	HT-29	Induction of apoptosis
[17]	*Lactobacillus acidophilus* CL1285 *Lactobacillus casei* LBC80R (in the presence of 5-FU)	LS513	40% ↑ apoptosis
[28]	*Lactobacillus acidophilus* 606	HT-29	Inhibited proliferation of tumor cells
[19]	*Bacillus polyfermenticus*	NMC460	↓ Cell colony formation in cancer cells (N/E on normal colonocytes)
[29]	*Enterococcus faecalis* CECT7121	LBC	Inhibited proliferation of tumor cells, Induction od apoptosis
[11]	*Lactobacillus rhamnosus* GG *Bifidobacterium lactis* Bb12	Caco-2	↑ Apoptosis
[30]	*Enterococcus faecium* RM11 *Lactobacillus fermentum* RM28	Caco-2	Cell proliferation: ↓ 21% ↓ 23%
[31]	*Lactobacillus reuteri* ATCC PTA 6475	KBM-5	↑ Apoptosis
[16]	*Lactobacillus rhamnosus* GG	Caco-2	↓ level of IL–8
[32]	*Bifidobacterium adolescentis* SPM0212	Caco-2, HT-29, SW480	↓ Cell proliferation
[14]	*Lactobacillus rhamnosus* GG	HGC-27	↓ Cell proliferation Induction of apoptosis
[18]	*Lactobacillus acidophilus* SNUL *Lactobacillus casei* YIT9029 *Bifidobacterium longum* HY8001	SNUC2A, SNU1, NIH/3T3 and Jurkat cell	Suppressed proliferation of tumor cells
[33]	*Propionibacterium acidopropionici* CNRZ80	HT-29	↓ Cell proliferation Induction of apoptosis

Abbreviations: ↓ Decrease; ↑ increase; N/E no effect; human colonic cancer cells: Caco-2, HT-29, SW1116, HCT116, SW480, DLD-1, LoVo; human colonic epithelial cells: NMC460; human colorectal carcinoma cells: LS513, SNUC2A; human gastric adenocarcinoma cells: AGS; human gastric carcinoma cells: SNU1; human myeloid leukemia-derived cells: MDR, KMB-5; human T lymphocyte cells: Jurkat cel; mus musculus colon carcinoma cells: CT26; HGC-27, HGT-1; mus musculus embryonic fibroblast: NIH/3T3; spontaneous murine T-cell lymphoma cells: LBC, 5FU5-Fluorouracil.

**Table 2:** Effects of probiotics on tumor-bearing or tumor-induced in animal models in vivo.

Reference	Probiotic Strain	Model	Induction	Time of Treatment	Effect
[36]	*Lactobacillus casei* BL23	C57BL/6 mice	DMH	10 weeks	↓ TI
[34]	*Pediococcus pentosaceus* GS4	Swiss albino mice	AOM	4 weeks	↓ TP Induction of apoptosis
[37]	*Lactobacillus rhamnosus* GG CGMCC 1.2134	SD rats	DMH 10 weeks	25 weeks	↓ TI ↓ TV ↓ TM Induction of apoptosis
[38]	*Lactobacillus acidophilus* *Bifidobacterium bifidum Bifidobacterium infantum*	SD rats	antibiotics DMH	23 weeks	↓ TI ↓ TV
[39]	*Lactobacillus salivarius* Ren	F344 rats	DMH 10 weeks	2 weeks ^a^	↓ TI
[40]	*Lactobacillus plantarum* (AdF10) *Lactobacillus rhamnosus* GG	SD rats	DMH 4 weeks	One of strains 12 weeks	↓ TI ↓ TV ↓ TM
[15]	VSL#3 (Probiotics mixture)	C57BL/6 mice	DSS	2 weeks ^a^	↓ TI ↓ dysplasia
[41]	*Lactobacillus plantarum*	BALB/c mice	CT26 cells injection	14 weeks	↓ TV Induction of necrosis
[23]	*Lactobacillus plantarum*	BALB/c mice	AOM, DSS	Nanosized/ Live bacteria 4 weeks	↓ TI cell cycle arrest Induction of apoptosis
[42]	*Lactobacillus rhamnosus* GG MTCC #1408 *Lactobacillus acidophilus* NCDC #1	SD rats	DMH	19 weeks ^a^	↓ TI ↓ TM
[43]	*Lactobacillus acidophilus* KFRI342	F344 rats	DMH	10 weeks	↓ ACF ↓ β-glucuronidase and β-glucosidase activity
[44]	*Lactobacillus rhamnosus* 231 (Lr 231)	rats	MNNG	5 weeks	↓ fecal activity of azoreductase and nitroreductase, ↓ GST ↑GSH
[45]	VSL#3 (Probiotics mixture)	SD rats	TNBS	10 weeks	None of the animals developed CRC
[46]	*Lactobacillus plantarum*	Wistar albino rats	DMH	6 weeks	↓ the activities of bacterial enzymes, the fecal bile acids concentration ↑ serum TNFα level
[19]	*Bacillus polyfermenticus*	CD-1 mice	DLD-1 cells injection	20 weeks (injection)	↓ TI ↓ TV
[28]	*Bifidobacterium lactis* KCTC 5727	SPF C57BL rat	–	19 weeks	↓ TI ↓ TV
[47]	*Lactobacillus acidophilus*, *Lactobacillus casei* *Lactobacillus lactis biovar diacetylactis* DRC-1	Rat	DMH	40 weeks	↓ TI ↓TV ↓ TM
[48]	*Bacillus polyfermenticus*	F344 rats	DMH	6 weeks	50% ↓ ACF, ↑ antioxidant potential

^a^ Before and until the end of experiment. Abbreviations: ↓ Decrease; ↑ increase; ACF—aberrant crypt foci; AOM—azoxymethane; CRC—colorectal cancer; DMH—1,2-dimethylhydrazine dihydrochloride; DSS—dextran sulfate sodium; GSH—glutathione; GST—glutathione S–transferase; MNNG—N–Methyl–N’–Nitro–Nitrosoguanidine; SD rat—Sprague–Dawley rat; TI—tumor incidence; TM—tumor multiplicity; TNBS—trinitrobenzene sulfonic acid; TNFα level—serum tumor necrosis factor-alpha; TP—tumor progression; TV—tumor volume.

**Table 3:** Examples of probiotic bacteria used in the chemoprevention and the anti-cancer therapy.

Reference	Type of Cancer	Strain/Strains
[49]	Colorectal carcinoma	*Bifidobacterium lactis* Bb12, *Lactobacillus rhamnosus* GG
[50]		*Bifidobacterium longum*, *Lactobacillus acidophilus, Enterococcus faecalis*
[51]		*Bacillus natto*, *Lactobacillus acidophilus*
[52]		*Lactobacillus plantarum* CGMMCC No 1258, *Lactobacillus acidophilus* LA-11, *Bifidobacterium longum* BL-88
[53]		*Lactobacillus rhamnosus* 573
[54]	Liver cancer	*Lactobacillus rhamnosus* LC705, *Propionibacterium freudenreichii* subsp. *shermanii*
[55]	Gastric cancer	*Lactobacillus reuteri* PTCC 1655
[56]		*Lactobacillus kefiri* P-IF
[57]	Cervical cancer	*Lactobacillus acidophilus*, *Bifidobacterium bifidum*

**Table 4:** Results of clinical studies using probiotic strains in the cancer prevention and the treatment.

Reference	Probiotic Strain	Subject	Time of Treatment	Effect
The Prevention				
[58]	Yakult containing *Lactobacillus casei* Shirota (LcS) and isoflavones from soy product	968 breast cancer patients aged 40 to 55.	2 years	↓ the incidence of breast cancer in Japanese women correlated with consumption of LcS and isoflavones since adolescence.
[59]	*Streptococcus thermophilus* and *Lactobacillus delbruckii* subsp. *bulgaricus*	45,241 healthy people (14,178 men, 31,063 women)	12 years	↓ the risk of colorectal cancer correlated with increased consumption of yogurt (especially in men).
[60]	*Lactobacillus gasseri* OLL2716 (LG21)	10 people with colorectal cancer and 20 healthy patients	12 weeks	↑ the number of bacteria from the genus *Lactobacillus*, ↑ synthesis of isobutyric acid, ↑ NK cell activity. ↓ the amount of *Clostridium perfringens*.
[19]	*Lactobacillus casei* Shirota (LcS)	54 women with an HPV-positive intra epithelial lesion	6 months	60 % ↓ in human papilloma virus (HPV) associated infection and cervical cancer precursors
[61]	*Lactobacillus rhamnosus* LC705, *Propionibacterium* *freudenreichii* ssp. *shermanii* JS	38 men (between 24 and 55 years old)	4 weeks	↓ β-glucosidase and urease activity ↑ amount of bacteria of the genus *Lactobacillus* and *Propionibacterium*
[54]	*Lactobacillus rhamnosus* LC705, *Propionibacterium freudenreichii* subsp. *shermanii*	90 male students with high aflatoxin level in urine	5 weeks	61.5% ↓ a liver cancer biomarker which leads to reduced urinary excretion of aflatoxin B1-N7guanine (AFB-N7-guanine)
[62]	*Lactobacillus acidophilus* L1	180 people with bladder cancer (mean age:67 years and 445 population-based controls	10 weeks	↓ the risk of bladder cancer correlated with habitual intake of lactic acid bacteria
The Treatment				
[63]	*Lactobacillus acidophilus*, *Lactobacillus plantarum, Bifidobacterium* *lactis*, *Saccharomyces* *boulardii*	164 patients with colorectal cancer undergoing colorectal surgery	30 days	↓ the risk of postoperative complications. In the probiotic group, a positive correlation was observed between the expression of the SOCS3 gene and the expression of the TNF gene and circulating IL–6.
[64]	*Lactobacillus plantarum* CGMCC, *Lactobacillus acidophilus*-11, *Bifidobacterium longum*-88	150 patients with colorectal cancer	6 days preoperatively and 10 days postoperative	↓ the serum zonulin concentration, ↓ duration of postoperative pyrexia, ↓ duration of antibiotic therapy, ↓ rate of postoperative infectious complications, Inhibited the p38 mitogen-activated protein kinase signaling pathway
[65]	*Bifidobacterium longum*	60 patients with colorectal cancer undergoing colon resection	3 days	↑ the count of bacteria of the genus *Bifidobacterium* ↓ the count of bacteria of the genus *Escherichia*
[66]	*Bifidobacterium breve* Yakult	42 patients during chemotherapy	6 weeks	↓ the incidence of fever ↓ the need for intravenous antibiotics using compared to the control group.
